# Ferrer Adjustable Speaking Valve for Early Phonation in a Deconditioned Patient

**DOI:** 10.7759/cureus.62081

**Published:** 2024-06-10

**Authors:** Rafael Cartaya, Syed A. A Rizvi, Viviane Manara, Mari L Tesch, Rahaghi Franck

**Affiliations:** 1 Family Medicine, Nova Medical Services, Miami, USA; 2 Biomedical Sciences, Larkin University, Miami, USA; 3 Pulmonary and Critical Care, Ferrer Pulmonary Institute, Hallandale Beach, USA; 4 Research and Development, Dr. Ferrer Biopharma, Hallandale, USA; 5 Pulmonary and Critical Care Medicine, Cleveland Clinic Florida, Weston, USA

**Keywords:** voice related quality of life, home health nursing, respiratory support, tracheostomy, speaking valve

## Abstract

A patient with multiple comorbidities and an eight-year history of tracheostomy was being treated for tracheitis. At this point, she became incapable of using regular speaking valves, and multiple attempts to reintroduce the speaking valve failed. A Ferrer adjustable speaking valve (FASV) was designed with gradations of outflow closure, allowing air to go through the vocal cords for phonation. The FASV was offered to her through the compassionate use program at the FDA. At 20% initial closure, the patient was able to tolerate the valve and was advanced to 50% closure, at which point she could phonate partially. The use of the valve was terminated at the time of her transfer, 23 days after the initiation of use. This suggests the safety and possible efficacy of using an adjustable speaking valve earlier than regular valves, allowing patients to communicate earlier and further exercise their diaphragms.

## Introduction

Patients with tracheostomy cannot phonate without some degree of tube occlusion during exhalation. This resistance is provided by a speaking valve, a one-way valve that provides minimal resistance during inspiration and shuts down for expiration, thus allowing phonation. Unfortunately, due to weakness, multiple tries are needed before the patient can tolerate this setup, as they would not be able to tolerate the full closure of the outflow path through the tracheostomy or even the additional resistance of the inflow path after the valve is added. Once the valve is in place, the patient can phonate with every breath if needed. Complete occlusion trials generally follow this in anticipation of tracheostomy discontinuation. A large body of evidence supports using speaking valves to improve communication in tracheostomy patients [1].

## Case presentation

Ms. J is a 46-year-old female with chronic respiratory failure s/p tracheostomy as well as a history of subglottic stenosis, restrictive lung disease 2/2 to severe kyphoscoliosis, Sjogren syndrome, antiphospholipid syndrome, systemic lupus erythematosus, major depression, and anxiety. She had a tracheostomy eight years prior.

She was being treated for possible pneumonia/tracheitis and finished a course of Cefepime. At that time, she could not tolerate multiple trials of a speaking valve. She self-occluded, by her finger, her tracheostomy site to communicate and make her needs known. Before starting the adjustable speaking valve trials, she was being treated for recurrent cellulitis of the right-sided peri-stomal tracheal area with an associated complicated boil. When she first started using the Ferrer adjustable speaking valve (FASV), she started at 20% occlusion and could only speak one word per breath. As her pulmonary status improved, the percent occlusion increased to 50%, and she improved to speak two words per breath consistently. She made continuous improvements as expected until the trials were placed on hold as she was preparing for transfer to another facility after 23 days of use. Of note was that the boil and cellulitis resolved and did not exacerbate while she continued with the Ferrer-adjustable speaking valve trials. No complications or adverse events were noted from the use of the FASV. In this case, the adjustable outflow obstruction allowed earlier use of a valve for phonation.

## Discussion

**Figure 1:**
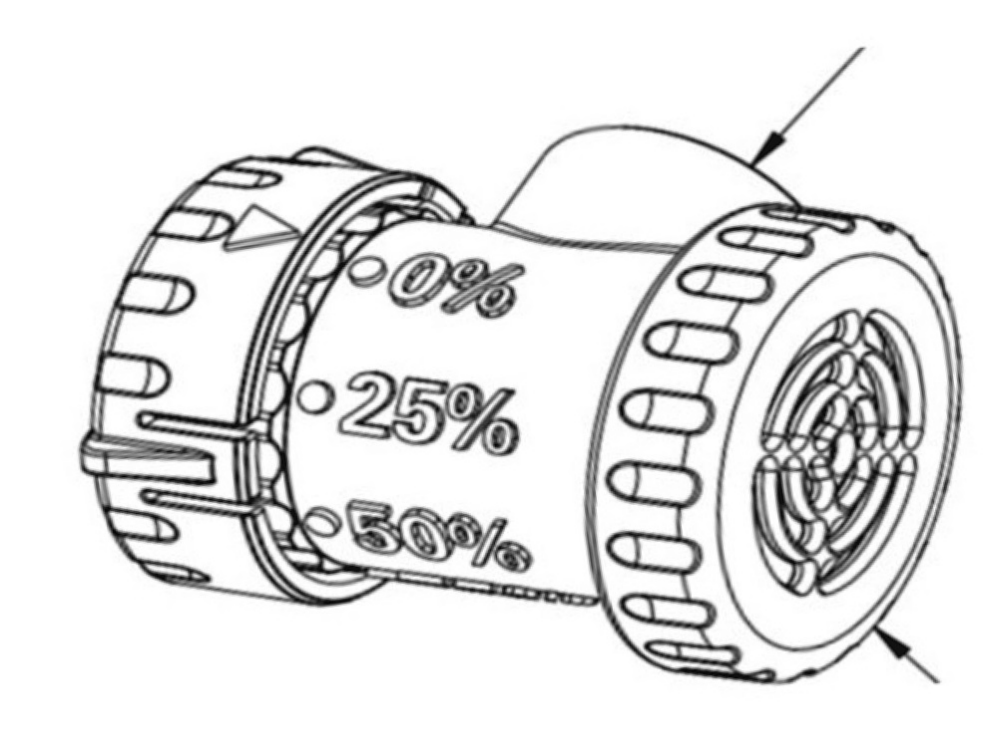
Ferrer adjustable speaking valve Long arrow: Attachment to patients tracheostomy hub. Short arrow: Inflow diaphragm. *Reprinted with permission of Dr. Ferrer Biopharma

The Ferrer Adjustable Speak Easy-Valve (FASV), made by Dr. Ferrer Biopharma, is a compact, lightweight apparatus engineered to fit the universal 15 mm hub of tracheostomy tubes (Figure 1). The FASV’s design includes a wider diameter pathway, preventing the restriction of inhaled air, a problem that may arise with other one-way speaking valves. The FASV can be used interchangeably by tracheostomized and ventilator-dependent patients. They can be easily adapted for use in line with a ventilator circuit and can be used alongside closed suctioning systems, swivel adapters, supplemental oxygen, and humidification.

A significant number of tracheostomized patients cannot tolerate the currently available speaking valves when they try them for the first time. Concurrently, about half of these patients require multiple trials before fully engaging with the device [2]. Studies indicate that most patients experience a feeling of suffocation when they first try the PMV [3]. In light of this, the FASV was designed to be set at an initial setting of 50% resistance/closure of the one-way outflow and meant to cap the valve to a complete 100% closure of the diaphragm. Once the one-way valve is closed, the outer part of the AV features an adjustable flow cylinder. The valve has been indexed for attenuating flow in 25% increments to 50%, 75%, and 100% (fully closed) using a knob instead of drilling holes (which can lead to inaccurate airflow) to improve tolerance [4,5].

Patients typically spend more than a week in the weaning process [6]. The FASV's initial benefit is that it enables earlier phonation. There is fair evidence that in-line speaking valves can accelerate phonation in those too weak for cannula deflation [7-9]. In addition, Martin et al. showed that placement of speaking valves within 24 hours is possible and safe [9,10]. The ultimate objective of FASV is to enhance patient satisfaction and reduce hospital length of stay by increasing diaphragmatic exertion and earlier successful decannulation.

The FASV is currently undergoing an FDA approval process. The FDA explicitly permitted the use of FASV in this patient under compassionate use.

## Conclusions

Regular speaking valves are initially hard to tolerate by deconditioned patients and those with significant respiratory insufficiency. An adjustable (resistance) speaking valve allowed the gradual introduction of phonation to a patient who could not tolerate regular speaking valves. This led to better communication, greater patient satisfaction, and perhaps further exercise of the patient’s diaphragm.
